# Publisher Correction: The low affinity neurotrophin receptor CD271 regulates phenotype switching in melanoma

**DOI:** 10.1038/s41467-018-02850-8

**Published:** 2018-01-22

**Authors:** Gaetana Restivo, Johanna Diener, Phil F. Cheng, Gregor Kiowski, Mario Bonalli, Thomas Biedermann, Ernst Reichmann, Mitchell P. Levesque, Reinhard Dummer, Lukas Sommer

**Affiliations:** 1 University of Zürich, Institute of Anatomy, Winterthurerstrasse 190, 8057 Zürich, Switzerland; 2 University of Zürich Hospital, Department of Dermatology, Gloriastrasse 31, 8091 Zürich, Switzerland; 3 University of Zürich Children’s Hospital, Tissue Biology Research Unit, August Forel Strasse 7, 8008 Zürich, Switzerland

Correction to: *Nature Communications* 10.1038/s41467-017-01573-6 (2017), published online 7 December 2017

The originally published version of this Article was updated shortly after publication to add the words ‘The’ and ‘affinity’ to the title, following their inadvertent removal during the production process. This has now been corrected in both the PDF and HTML versions of the Article.

