# Supplementary material for: The low affinity neurotrophin receptor CD271 regulates phenotype switching in melanoma
Source: Nat Commun. 2017 Dec 7;8:1988. doi: 10.1038/s41467-017-01573-6 (PMC5719420; doi:10.1038/s41467-017-01573-6)
Supplement: Supplementary file 1 — Supplementary Information [file 41467_2017_1573_MOESM1_ESM.pdf]

The low affinity neurotrophin receptor CD271 regulates phenotype switching in melanoma

Supplementary Information

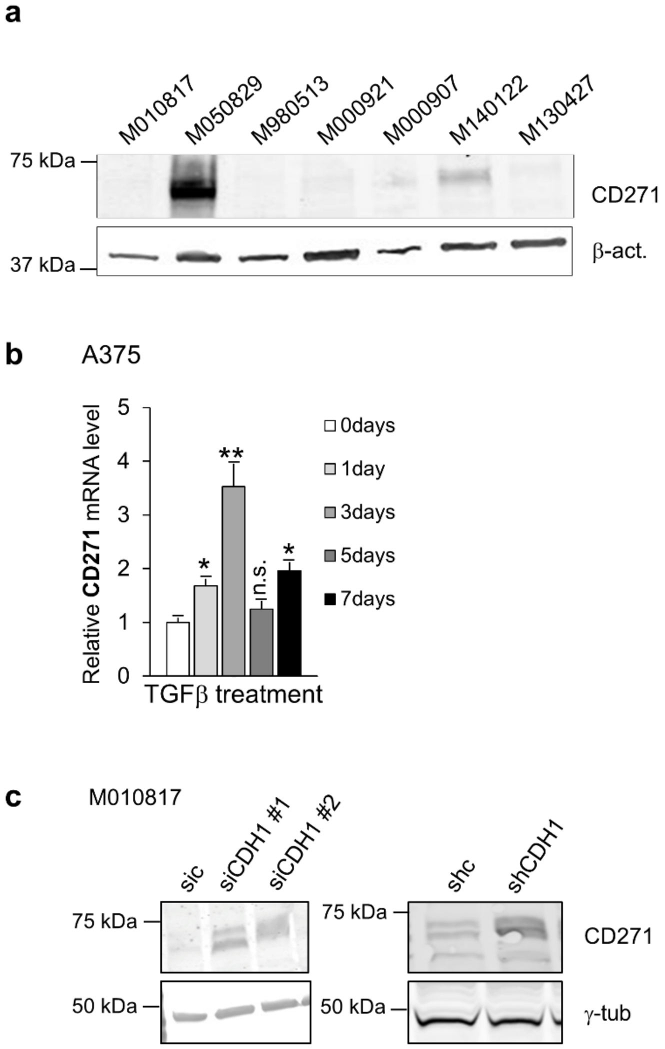

Supplementary Figure 1: CD271 expression correlates with an invasive signature (**a**) Western blot of different melanoma cell lines for CD271 protein. (**b**) qRT-PCR for CD271 mRNA in the melanoma cell line A375 treated with TGFβ1 (2ng/ml) at different time points (n = 3, P value ≤ 0.05). Error bars indicate S.D. (**c**) Western blot for CD271 protein in cells with transient or stable knock down of CDH1 (two different siCDH1 were used and one shCDH1. See Supplementary Table 4).

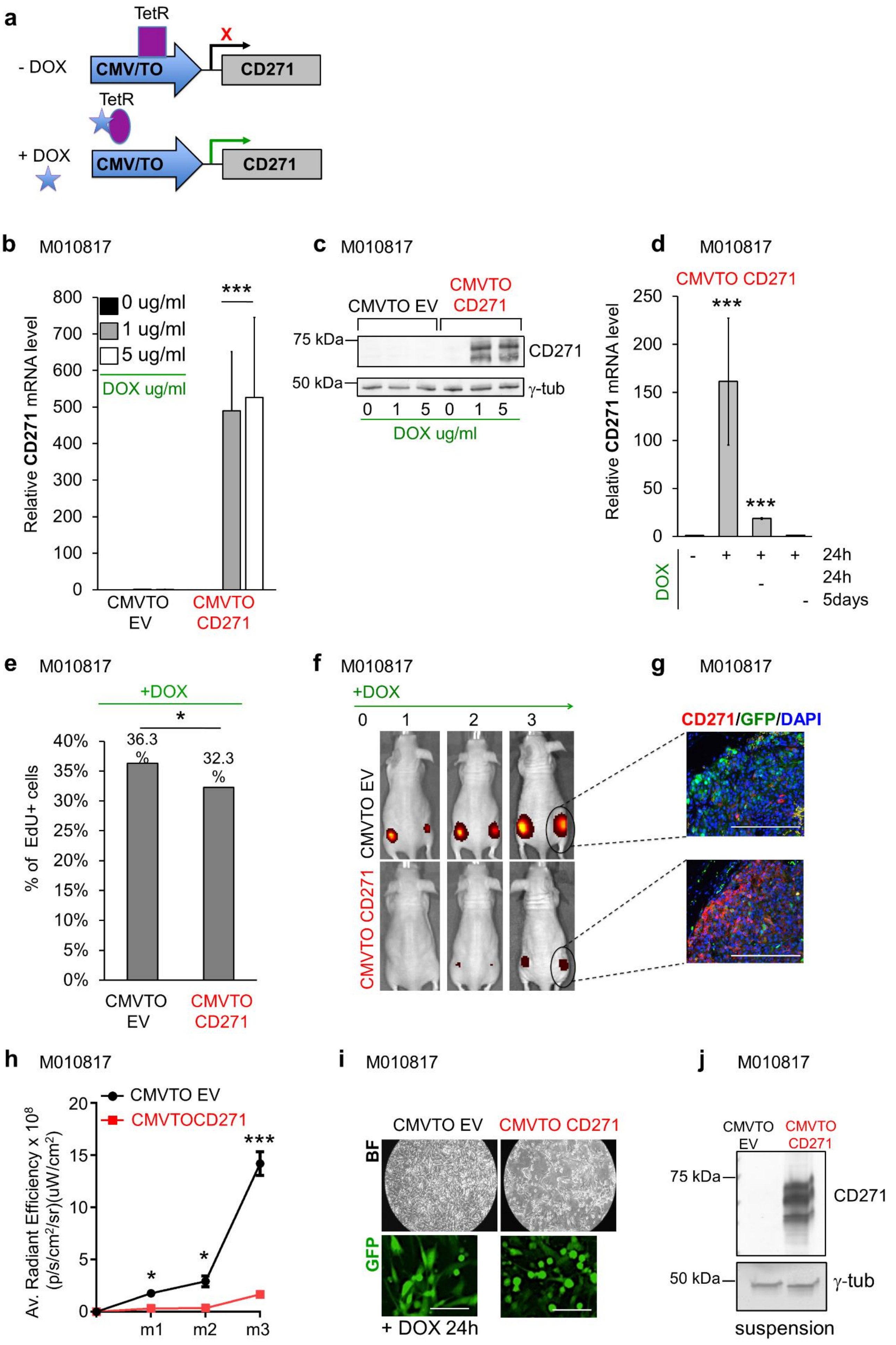
a
TetR
- DOX
CMV/TO
CD271
TetR
+ DOX
CMV/TO
CD271
b M010817
0 ug/ml
1 ug/ml
5 ug/ml
DOX ug/ml
***
Relative CD271 mRNA level
CMVTO EV
CMVTO CD271
c M010817
CMVTO EV
CMVTO CD271
75 kDa
50 kDa
CD271
γ-tub
0 1 5 0 1 5
DOX ug/ml
d M010817
CMVTO CD271
***
***
Relative CD271 mRNA level
DOX
24h
24h
5days
e M010817
+DOX
*
36.3 %
32.3 %
% of EdU+ cells
CMVTO EV
CMVTO CD271
f M010817
+DOX
0 1 2 3
CMVTO EV
CMVTO CD271
g M010817
CD271/GFP/DAPI
h M010817
CMVTO EV
CMVTOCD271
*
*
***
Av. Radiant Efficiency x 10^8 (p/s/cm²/sr)(uW/cm²)
m1 m2 m3
i M010817
CMVTO EV
CMVTO CD271
BF
GFP
+ DOX 24h
j M010817
CMVTO EV
CMVTO CD271
75 kDa
50 kDa
CD271
γ-tub
suspension

Supplementary Figure 2: Doxycycline-inducible CD271 expression

(**a**) Representation of the TetON system based on the TRex™ technology used for the switch experiment in Figure 4[1]. In the absence of doxycycline, the Tet Repressor (TetR) protein binds to the Tet Operon (TO), inhibiting transcription of the target gene (CD271). Upon addition of doxycycline, which can bind to TetR, TetR undergoes a conformation change that leads to the detachment from the TO and the transcription of the target gene from the CMV promoter. (**b, c**) CD271 expression in M010817 cells was assessed by qRT-PCR and western blot after doxycycline administration at different concentrations in cells carrying the CMVTOEV or the CMVTOCD271 vectors (n = 3, P value ≤ 0.001). Error bars indicate S.D. (**d**) qRT-PCR of CMVTOCD271 cells treated with doxycycline and then released for 1 or 5 days to show the switch in expression of the gene (n = 3, P value ≤ 0.001, P value > 0.05). Error bars indicate S.D. (**e**) FACS analysis for EdU incorporation (n = 3; P value ≤ 0.05). (**f**) *In vivo* imaging of Nude mice injected with cells carrying CMVTOEV or CMVTOCD271 vectors constantly treated with doxycycline for 3 weeks. (**g**) Immunofluorescence of CD271 in xenografts obtained from mice in (f). Scale bars 200 µm. (**h**) Quantification of IVIS signal for iRFP (4 mice for a total of 8 injections were analyzed for each conditions; P value_m1 ≤ 0.05, P value_m2 ≤ 0.05, P value_m3 ≤ 0.001). Error bars indicate S.E.M. (**i**) Brightfield (upper panel) and fluorescent micrographs (lower panel) of CMVTOEV or CMVTOCD271 melanoma cells treated with doxycycline for 24h. Scale bars 50 µm. (**j**) Western blot for CD271 protein of cells (either infected with CMVTOEV or CMVTOCD271 vectors) in suspension after doxycycline treatment for 24h.

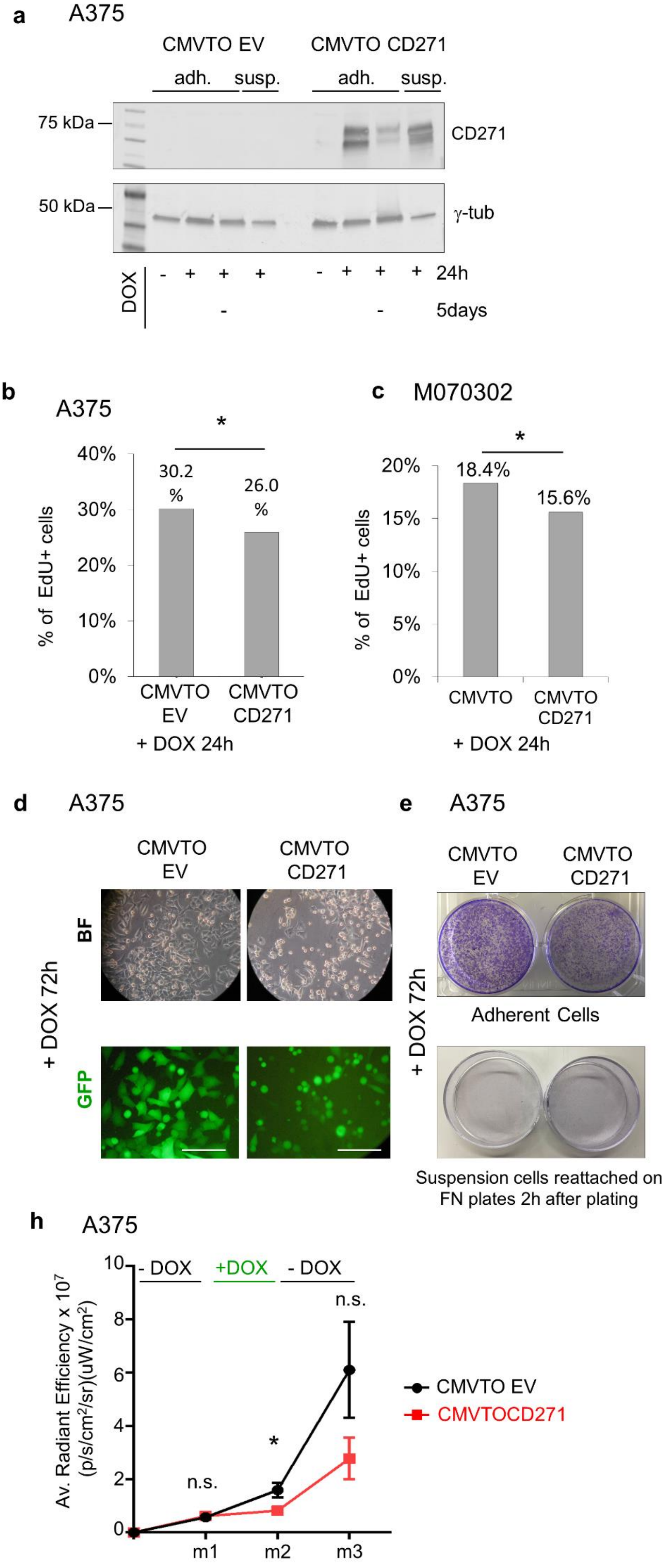
a
A375
CMVTO EV
CMVTO CD271
adh.
susp.
adh.
susp.
75 kDa
CD271
50 kDa
γ-tub
DOX
- + + + - + + + 24h
- - 5days
b
A375
*
40%
30%
20%
10%
0%
30.2 %
26.0 %
% of EdU+ cells
CMVTO EV
CMVTO CD271
+ DOX 24h
c
M070302
*
20%
15%
10%
5%
0%
18.4%
15.6%
% of EdU+ cells
CMVTO
CMVTO CD271
+ DOX 24h
d
A375
CMVTO EV
CMVTO CD271
BF
GFP
+ DOX 72h
e
A375
CMVTO EV
CMVTO CD271
+ DOX 72h
Adherent Cells
Suspension cells reattached on FN plates 2h after plating
f
A375
Relative amount of cells in suspension
*
CMVTO EV
CMVTO CD271
+ DOX 72h
g
M070302
Relative amount of cells in suspension
**
CMVTO EV
CMVTO CD271
+ DOX 48h
h
A375
- DOX
+DOX
- DOX
n.s.
*
n.s.
Av. Radiant Efficiency x 10^7 (p/s/cm²/sr)(uW/cm²)
m1
m2
m3
CMVTO EV
CMVTOCD271

Supplementary Figure 3: Transient CD271 expression in cell lines A375 and M070302

(**a**) Western Blot of adherent and suspension cell fractions of A375 cells carrying either CMVTOEV or CMVTOCD271 constructs and treated with doxycycline (1 μg/ml). (**b**) Measurement of EdU incorporation (30 min pulse) in A375 CMVTOEV and CMVTOCD271 cells after 24h treatment with doxycycline. (**c**) Measurement of EdU incorporation (30 min pulse) in M070302 CMVTOEV and CMVTOCD271 cells after 24h treatment (1 μg/ml) with doxycycline. (**d**) Brightfield and fluorescence micrographs of A375 CMVTOEV and CMVTOCD271 after 72h of doxycycline treatment (1 μg/ml). Scale bars 50 μm. (**e**) Crystal violet staining of adherent and suspension (reattached on FN plates 2h after plating) A375 CMVTOEV and CMVTOCD271 cells after 72h of doxycycline treatment (1 μg/ml). (**f**) Quantification of A375 CMVTOEV and CMVTOCD271 cells in suspension after 72h of doxycycline treatment. (**g**) Quantification of M070302 CMVTOEV and CMVTOCD271 cells in suspension after 72h doxycycline treatment. Error bars for (f) and (g) indicate S.D. (**h**) Quantification of the iRFP signal by IVIS in nude mice injected with A375 CMVTOEV and CMVTOCD27. m1 = before doxycycline treatment; m2 = during doxycycline treatment; m3 = after release from doxycycline (6 mice for a total of 12 injections were analyzed for both conditions; P value_m1 > 0.05, P value_m2 ≤ 0.05, P value_m3 > 0.05). Error bars indicate S.E.M.

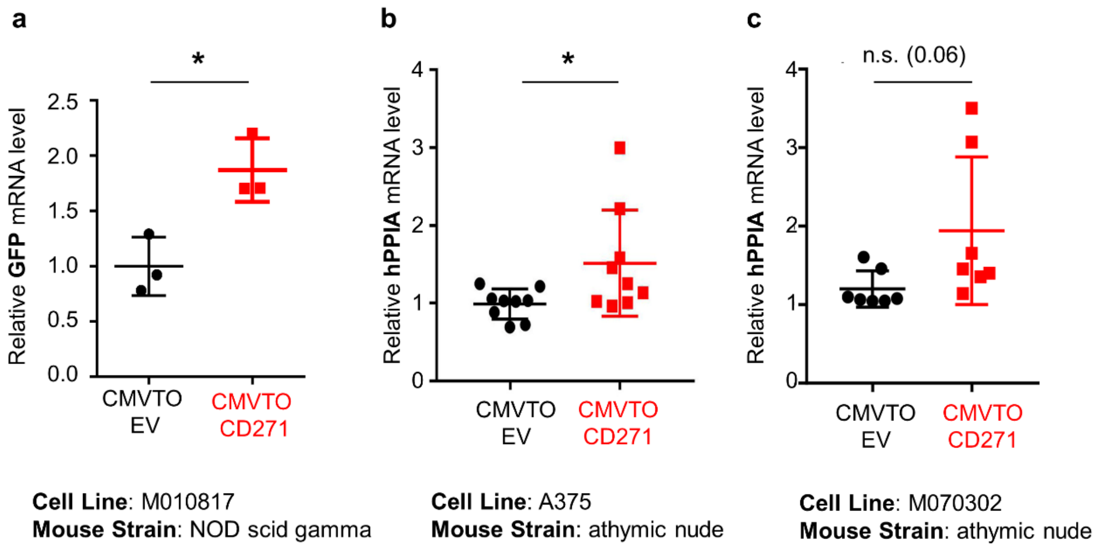

Supplementary Figure 4: Lung metastasis after transient CD271 expression
(**a**) qRT-PCR for GFP transcripts in lung lysates of mice from an experiment designed as in Figure 4a (switching CD271 expression) but done with NSG instead of Nude mice. N = 3, P value ≤ 0.05. (**b**) qRT-PCR for human PPIA transcripts in lung lysates of mice from an experiment designed as in Figure 4a but with the cell line A375. Human PPIA levels were measured instead of GFP because GFP levels were remarkably lower in A375 than in M010817. N = 9, P value ≤ 0.05. (**c**) qRT-PCR for human PPIA transcripts in lung lysates of mice from an experiment designed as in Figure 4a but with the cell line M070302. Human PPIA levels were measured instead of GFP because GFP levels were remarkably lower in M070302 than in M010817. N = 7, P value > 0.05. All error bars indicate S.E.M.

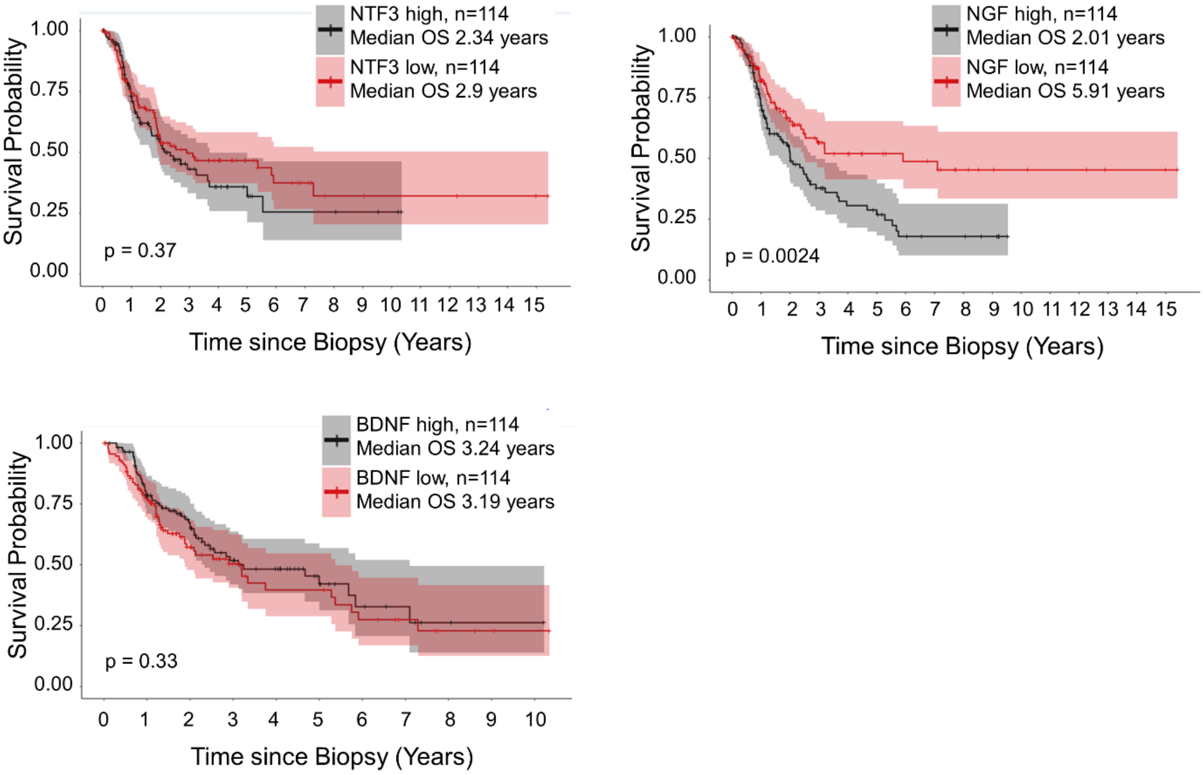

Supplementary Figure 5: Patient survival curves in respect of neurotrophins
Survival curves based on TCGA data for expression of the neurotrophins NT3, BDNF and NGF in human melanoma samples. Data from 114 patients per condition were analyzed, which represent 25% of highest and lowest expressing patients.

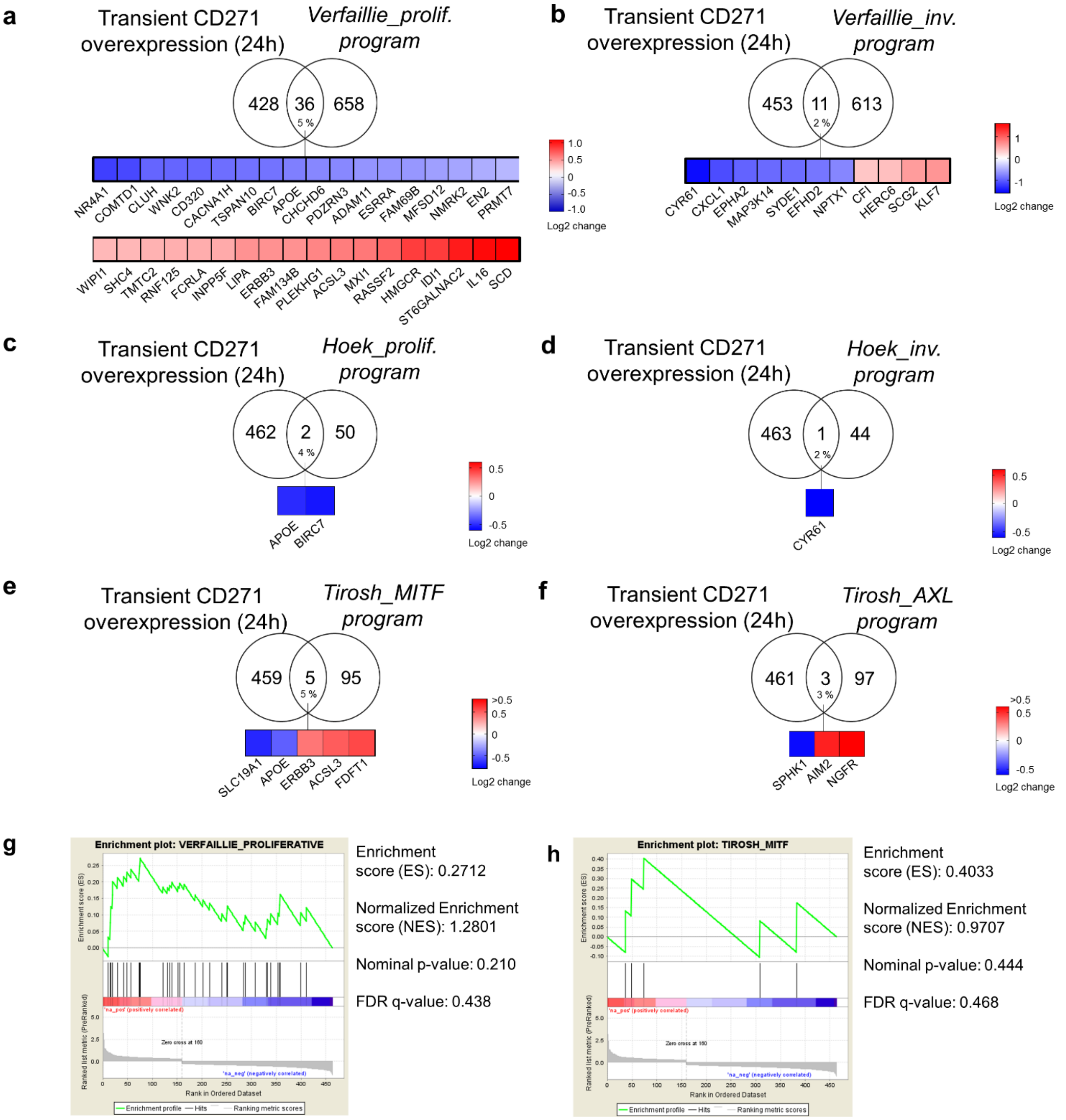

Supplementary Figure 6: Comparing transient CD271 overexpression signature with established invasive/proliferative signatures

Venn diagrams (http://bioinfogp.cnb.csic.es/tools/venny/index.html) for comparison of transient CD271 overexpression RNA Seq data with Verfaillie proliferative (**a**) and invasive (**b**) programs, with Hoek proliferative (**c**) and invasive (**d**) programs and with Tirosh MITF (**e**) and AXL programs (**f**) programs. All gene expression heatmaps represent significant Log2 values obtained from RNA Seq. The following cut-offs were applied for transient CD271 overexpression: Log2 ≤-0.27 or ≥0.27, *P* value <0.05, FDR <0.05. The Verfaillie and Hoek gene signatures/gene lists were obtained from Falletta et al.[2]. The Tirosh MITF and AXL programs were obtained from Tirosh et al.[3]. For Venn diagrams with an overlap of the transient CD271 overexpression program with at least 5 % of the respective programs of interest, GSEA was run with 1000 permutations. (**g**) GSEA of Suppl. Fig. 6a. (**h**) GSEA of Suppl. Fig. 6e.

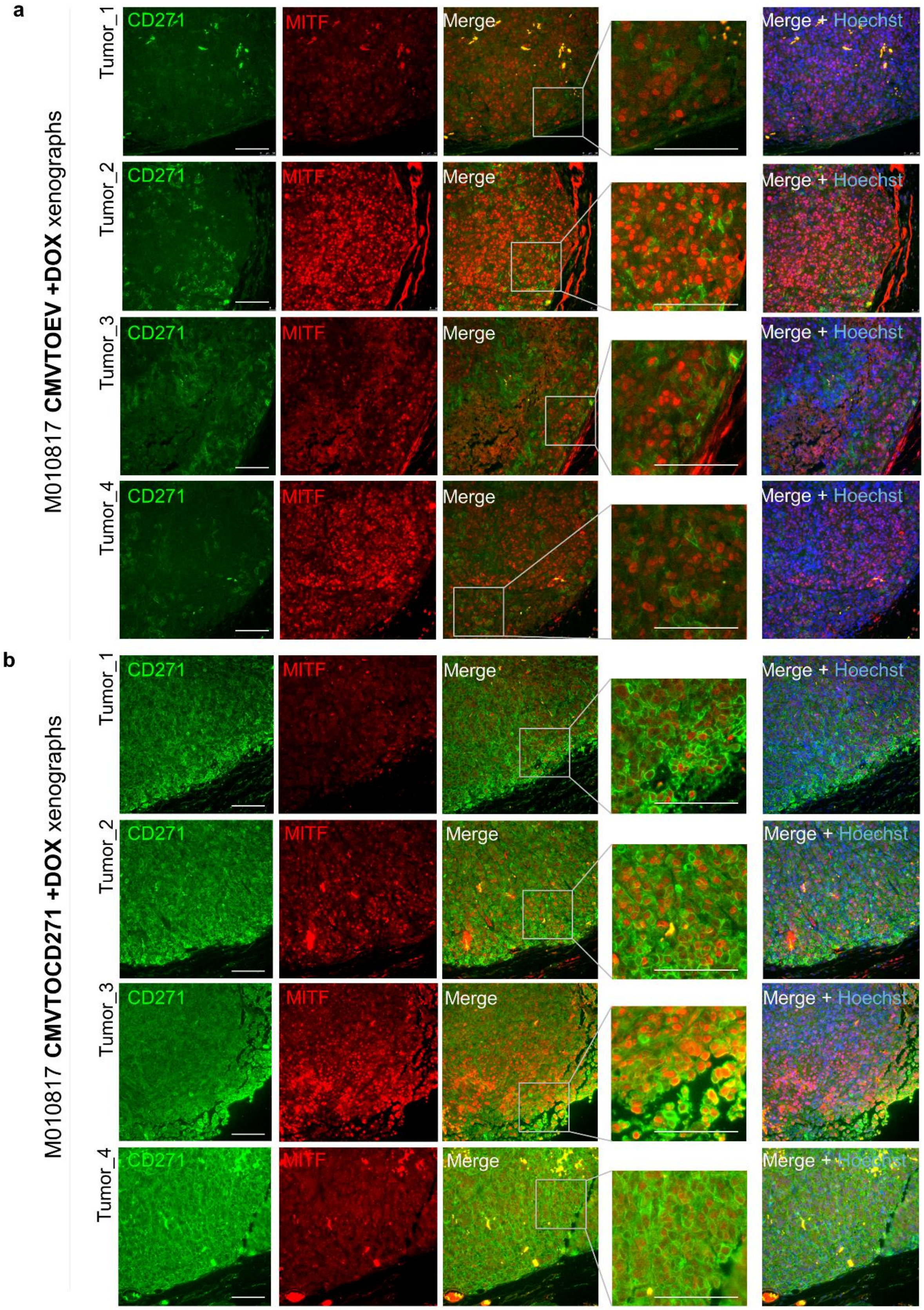

Supplementary Figure 7: MITF expression in xenographs

Immunofluorescent stainings of xenograft tumors from M010817 cells either carrying CMVTOEV (**a**) or CMVTOCD271 (**b**) constructs. One week after subcutaneous cell injection, doxycycline was administered to mice through drinking water until euthanasia. Scale bars 100 µm.

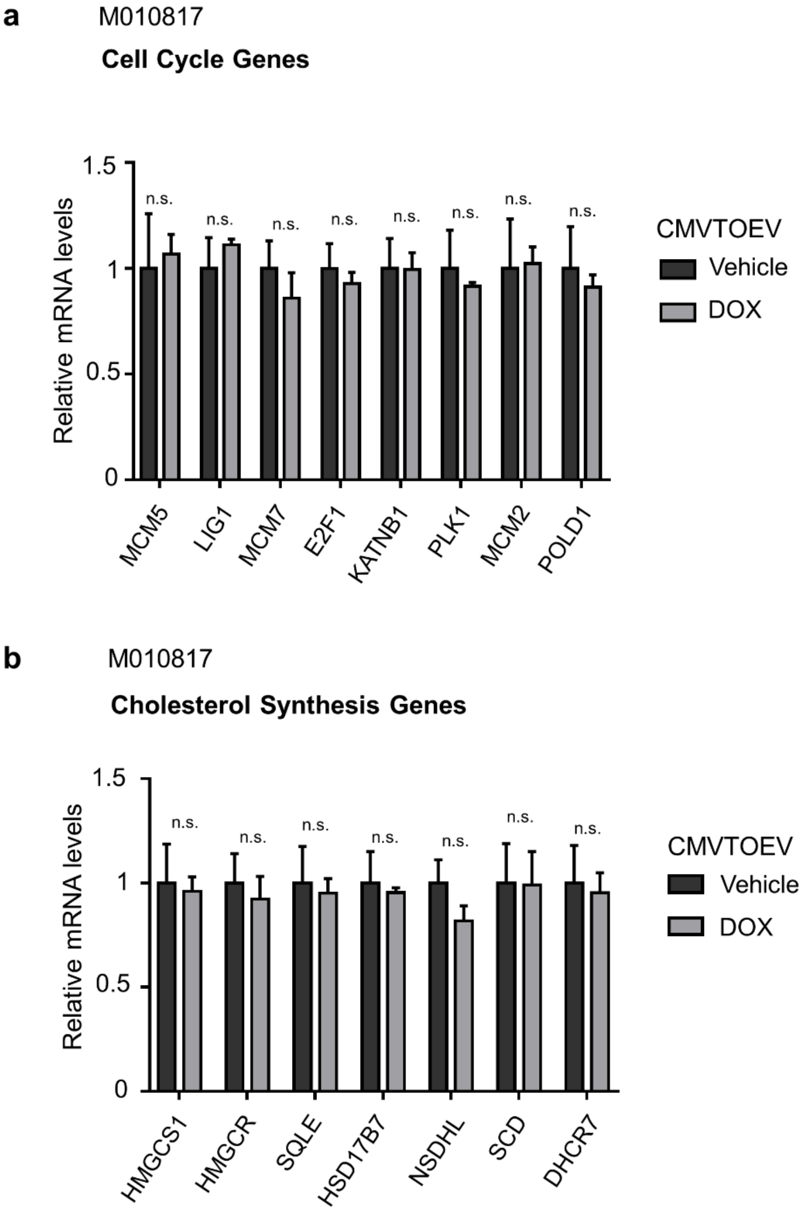

Supplementary Figure 8: Doxycycline controls

(**a**) qRT-PCR for specific cell cycle genes in M010817 CMVTOEV cells either treated with vehicle or with doxycycline (1 μg/ml) for 24h. (**b**) qRT-PCR for specific cholesterol and lipid synthesis genes in M010817 CMVTOEV cells either treated with vehicle or with doxycycline (1 μg/ml) for 24h. For (a) and (b), n = 3, P value ≥ 0.05. All error bars indicate S.D.

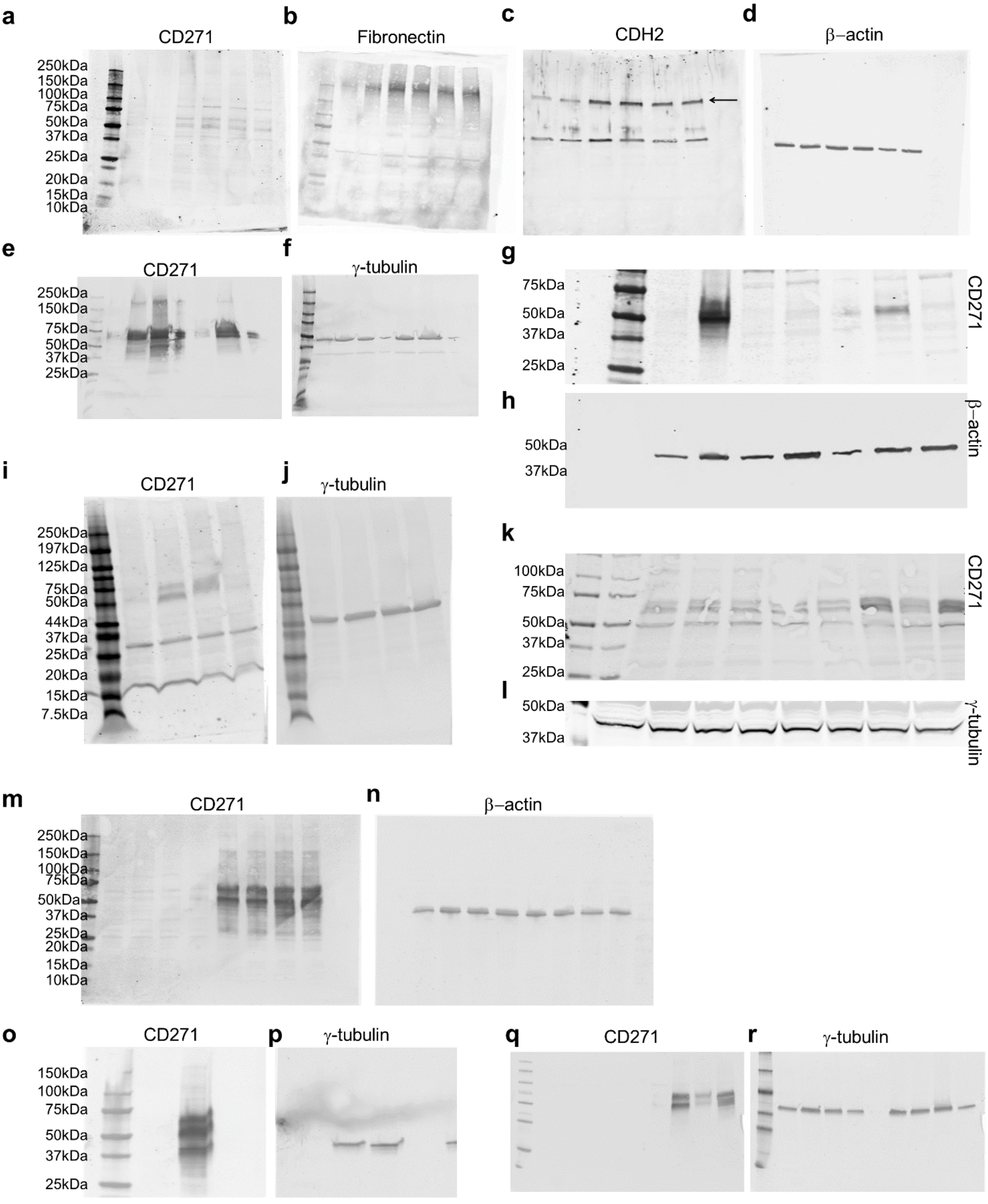

Supplementary Figure 9: Western Blot originals from main figures
Original western blots shown in the different figures. (**a-d**) Figure 2e. (**e,f**) Figure 3i. (**g,h**) Supplementary Figure 1a. (**i-l**) Supplementary Figure 1c. (**m,n**) Supplementary Figure 2c (**o,p**) Supplementary Figure 2j (**q,r**)Supplementary Figure 3a.

Supplementary Table 1: Primers for qRT-PCR

Target	Forward Sequence	Reverse Sequence
β-Actin	GGACTTCGAGCAAGAGATGG	AGGAAGGAAGGCTGGAAGAG
GADPH	ACCCAGAAGACTGTGGATGG	TCTAGACGGCAGGTCAGGTC
GFP	TCGAGCTGGACGGCGACGTA	ATGTGGTCGGGGTAGCGGC
CD271	CCTACGGCTACTACCAGG ATG	CACACGGTGTTCTGCTTGT
FN1	CAGTGGGAGACCTCGAGAAG	TCCCTCGGAACATCAGAAAC
CDH2	ACAGTGGCCACCTACAAAGG	CCGAGATGGGGTTGATAATG
Trk-A	GTCAGCCACGGTGATGAAATC	CAGCACGTCACGTTCTTCCT
MITF	GCCTCCAAGCCTCCGATAAG	CATCTGCTCACGCATGAGTTG
HMGCS1	GGCTGCCACTCTGTACTCTCTTA	AGGGAGTCTTGGTACTTTCTTGG
HMGCR	TGTGGCCAGCACCAATAGAG	CAACTGGGCCACGAGTCAT
GGPS1	AAGCACCCAGGTGCAGAATA	CATCTGCTCACGCATGAGTTG
SQLE	TATGGCAGAGCCCAATGCAA	AGCCCATCTGCAACAACAGT
NSDHL	TCGCACGGACTCATTTGACA	GGTGACGCACAGTGGAAAAC
HSD17B7	TCATCTCGCAGTGCAAGGAA	GAGACCCTGCTGGTTGAAGT
DHCR7	CGGATCGGGAAGTGGTTTGA	CAGACCCTGCAGCGTGTAAA
SCD	ACGCTTGTGCCCTGGTATTT	ACCAGCCAGGTGGCATTAAG
E2F1	CGTGTCAGGACCTTCGTAGC	CATCGATCGGGCCTTGTTTG
KATNB1	ACATCAAGACGTCGGTGGAC	TCTCAATCTGTGGCAGGACG
LIG1	ATGGACAGTTCCCCATCAGG	TCTGCTTTGGAGGTCTTTAGGG
MCM2	ACATCGAGTCCATGATCCGC	CAGCTCATTGTTGTCACGCC
MCM5	AGCCCTTCCAGCATTCGTAG	GCATGGCAATGTTGGTGAGG
MCM7	GCTCTGGCACGTCTGAGAAT	ACGGACGGTGGCAAATATCA
PLK1	GCTTTGCCAAGTGCTTCGAG	AATCCTACGACGTGCTGGTG
POLD1	CATGGGAACTCCTAGCCCTGA	TCAGTCAGGCCCAGTTTCTG
FDPS	CGGGTGAAGGCGCTATATGA	TCTCCGCTTGTAGATTTTGCG
CD271 ICD	GCCTTCAAGAGGTGGAACAGCTG	TGTCCACGGAGATGCCACTGTC

Supplementary Table 2: Information about Cell Lines used in experiments

Cell line	Patient Gender	Excision Location	Known Mutation	Origin/Vendor
M070413	M	Lymph node	Unknown	University Hospital Zurich, Department of Dermatology
M010817	F	Skin	$NRAS^{Q61R}$	University Hospital Zurich, Department of Dermatology
M070302	F	Lung	Unknown	University Hospital Zurich, Department of Dermatology
A375	F	Skin	$BRAF^{V600E}$	ATCC
M050829	F	Retro-Auricular	$NRAS^{Q61L}$	University Hospital Zurich, Department of Dermatology

Cell line	Treatment
M070413	Immune therapy with CP-675, 206 (CTLA4), Pfizer study; radiation, Chemotherapy with Temoda
M010817	radiation; immune therapy with interferon; vaccination with tumour-lysate pulsed DC; refreshing of vaccination; 2x refreshing of vaccination, CR
M070302	Pegasys; therapy with Dacarbazin and Thalidomid (6 cycles) ; immune-modulated therapy (3M-study) ; chemotherapy foursome combination with Velcade; chemotherapy VP-scheme (Eldisine, Platinol, Nexavar, Pegasys)
A375	
M050829	percutaneous radiation

Supplementary Table 3: Antibodies for immunofluorescence

Primary Antibody	Host	Company	Cat. No.	Dilution
CASPASE-3 CLEAVED	Rabbit	Cell Signaling	9661	1:200
GFP	Chicken	Aves	GFP-1020	1:400
CD271 ($p75^{NTR}$)	Rabbit	Alomone Labs	ANT-007	1:200
SOX10	Goat	Santa Cruz	Sc-17342	1:200
MITF	Rabbit	Gift from H. Arnheiter's lab	Ref[4,5]	1:200

Supplementary Table 4: siRNA, shRNA

Gene	Company	References
siNGFR	Life Technology (Invitrogen)	NGFRHSS107179<br>NGFRHSS181525
siCDH1	Life Technology (Invitrogen)	CDH1HSS101669<br>CDH1HSS101670
siTrk-A	Life Technology (Invitrogen)	TrkAHSS107335<br>TrkAHSS107336
shCDH1	Addgene	pLKO.1 puro shRNA CDH1 (#18801)
shCD271	Sigma-Aldrich(sequence from TRC Broad Institute)	pLKO.1 puro shRNA CD271 (#TRCN0000058154)
shcontrol	Addgene	pLKO.1 puro scrambled shRNA (#1864)

Supplementary Table 5: Antibodies for western blot

**Primary Antibody**	**Host**	**Company**	**Cat. No.**	**Dilution**
β-Actin	Mouse	Sigma Aldrich	A5316	1:10000
γ-Tubulin	Mouse	Sigma Aldrich	T-6557	1:10000
FIBRONECTIN	Rabbit	Abcam	F3648	1:300
CDH2	Rabbit	Lab Force	sc-7939	1:3000
CD271 ($p75^{NTR}$)	Rabbit	Alomone Labs	ANT-007	1:300
SOX10	Goat	Santa Cruz	sc-17342	1:1000
**Secondary Antibody**	**Host**	**Company**	**Cat. No.**	**Dilution**
IRDye680LT anti-mouse	Donkey	Li-Cor	926-68022	1:10000
IRDye800CWanti-mouse	Donkey	Li-Cor	926-32212	1:10000
IRDye680LT anti-rabbit	Donkey	Li-Cor	926-68023	1:10000
IRDye680LT anti-goat	Donkey	Li-Cor	926-68074	1:10000

Supplementary References

1. Yao, F. *et al.* Tetracycline repressor, tetR, rather than the tetR-mammalian cell transcription factor fusion derivatives, regulates inducible gene expression in mammalian cells. *Hum. Gene Ther.* **9,** 1939–1950 (1998).
2. Falletta, P. *et al.* Translation reprogramming is an evolutionarily conserved driver of phenotypic plasticity and therapeutic resistance in melanoma. *Genes Dev.* **31,** 18–33 (2017).
3. Tirosh, I. *et al.* Dissecting the multicellular ecosystem of metastatic melanoma by single-cell RNA-seq. *Science* **352,** 189–96 (2016).
4. Opdecamp, K. *et al.* Melanocyte development in vivo and in neural crest cell cultures: crucial dependence on the Mitf basic-helix-loop-helix-zipper transcription factor. *Development* **124,** 2377–2386 (1997).
5. Bharti, K., Liu, W., Csermely, T., Bertuzzi, S. & Arnheiter, H. Alternative promoter use in eye development: the complex role and regulation of the transcription factor MITF. *Development* **135,** 1169–78 (2008).
