# Supplementary material for: The low affinity neurotrophin receptor CD271 regulates phenotype switching in melanoma
Source: Nat Commun. 2017 Dec 7;8:1988. doi: 10.1038/s41467-017-01573-6 (PMC5719420; doi:10.1038/s41467-017-01573-6)
Supplement: Supplementary file 3 — Description of Additional Supplementary Files [file 41467_2017_1573_MOESM3_ESM.pdf]

Description of Additional Supplementary Files

File Name: Supplementary Data 1
Description: Differentially expressed genes Microarray Figure 1

File Name: Supplementary Data 2
Description: Differentially expressed genes RNA Seq Figure 7
